# Remittances for 1878

**Published:** 1877-11-20

**Authors:** 


					﻿Editorial and Miscellaneous.
Remittances for 1878.—We shall expect remit-
tances for 1878 to be made on or before, if possible
tbe reception of the January nuumber. Hope as
many as can, will remit in the month of Decem-
ber. If all would do so it would greatly facilli-
tate our arrangements for the ensuing year.
Unpaid subscriptions for 1877 are most urgent-
ly needed.
				

